# Evaluation and prediction of carbon emission from logistics at city scale for low-carbon development strategy

**DOI:** 10.1371/journal.pone.0298206

**Published:** 2024-02-29

**Authors:** Junyu Chen, Yan Zhu, Chuanming Yang, Huimin Wang, Ke Wang

**Affiliations:** 1 School of Business, Suzhou University of Science and Technology, Suzhou, Jiangsu Province, China; 2 College of Management and Economics, Tianjin University, Tianjin, China; 3 Business School, Hohai University, Nanjing, Jiangsu Province, China; Qufu Normal University, CHINA

## Abstract

Low-carbon is a part of China’s efforts to pursue the national strategy of “carbon peaking and carbon neutrality.” Meanwhile, the path of low-carbon transformation of logistics has become a topic of global concern. This study constructs a technical framework of logistics carbon emissions (LCE), which is composed of carbon emission evaluation, carbon emission prediction and low-carbon strategy. All 13 prefecture-level cities in Jiangsu, China, are the application objects in empirical research. Then, the influence analysis of the LCE efficiency based on the panel Tobit model and the evolution of LCE under different scenarios are explored. The results show that: (ⅰ) during the study period (2013–2020), the LCE in Jiangsu showed an overall upward trend, with Xuzhou, Suzhou and Nanjing being the cities with the highest carbon emissions; (ⅱ) the static efficiency of LCE in Jiangsu is at a medium level, with fluctuations in Suzhou, Changzhou, Zhenjiang, Nantong, and Suqian caused by the technical change index; (ⅲ) economic level, industrial structure, fixed asset utilization rate, and ecological environment in Jiangsu are significantly positively correlated with LCE efficiency, while education popularization and energy intensity are negative; (ⅳ) LCE in Jiangsu has been drastically reduced in the low-carbon scenario compared to the baseline scenario. On the above basis, this study proposes suggestions for the low-carbon development strategies of logistics in Jiangsu.

## 1. Introduction

China has entered a new stage of implementing the “carbon peaking and carbon neutrality goals”. At present, all industries have begun the low-carbon transformation, the existing literature mainly focus on low carbon development concepts, carbon emission assessment, digital technology applications, low carbon production benefits [1–4], etc. Logistics is a strategic and fundamental industry that supports the national economic activities, which should pay more attention to the green development process. Since 2020, China’s total social logistics growth rate has been higher than that of its gross domestic product (GDP), and the scale of logistics demand has grown steadily. In 2022, China’s total logistics revenue accounted for 10.49% of GDP, and the logistics market scale ranked first in the world for seven consecutive years. In 2020, the world’s CO_2_ emissions will be 319.8×10^8^ tons, and China’s CO_2_ emissions will be 98.92×10^8^ tons, accounting for 30.93% of the world’s, making China the largest contributor of carbon emissions. More seriously, energy consumption and LCE in China continue to grow. According to the statistics from the *Green Logistics Branch of the China Federation of Logistics*, the total energy consumption of China’s logistics was 3.85×10^8^ tons of standard coal in 2020, which accounted for 7.73% of total consumption, and the CO_2_ emissions of the whole industry were 8.57×10^8^ tons, accounting for 8.66% of total emissions. The low-carbon transformation of logistics is motivated by an urgent need for ecological protection, production efficiency improvement, and sustainable development.

Carbon emissions accounting is a key prerequisite for decision-making regarding low-carbon strategies. LCE refers to the emissions of carbon dioxide generated in the logistics process. The logistics mainly includes cargo transportation, warehousing, loading and unloading, and packaging, which consume large amount of energy and release greenhouse gases. Existing studies mainly focus on energy activities, industrial processes and product use, land use/land cover, and waste disposal [5–8]. Furthermore, practical measurements, mass balance methods, carbon emission factor methods, carbon footprint analyses, material flow analyses, and life cycle assessments have also been applied [9–13]. Scientific research institutions around the world, including the Global Carbon Budget, Emissions Database for Global Atmospheric Research, and Multi-resolution Emission Inventory for China and China Emission Accounts and Datasets. Additionally, carbon compensation policies, trade carbon deficit, land use/land cover pattern optimization, agricultural production mode, and urban architectural design [14–19], were discussed to explore the concept of carbon balance.

Evaluation of LCE performance is generally divided into two categories: (ⅰ) constructing the evaluation index system [20] and (ⅱ) measuring the efficiency value [21]. Carbon emission efficiency (CEE) measures the level of regional economic, social or industrial development under carbon emission constraints, and reflects the relationship between carbon emissions and economic growth. CEE can be considered from a single-factor and total-factor economic perspective. It can be evaluated by nonparametric methods (e.g., Data Envelopment Analysis, DEA) and parametric methods (e.g., Stochastic Frontier Analysis, SFA) [22]. While there are drawbacks to SFA in a multi-input-output scenario, DEA can compensate for these deficiencies. DEA models are divided into normal and super-efficiency models [23]. BCC, CCR, DDF, SBM, EBM, super-efficiency DEA [24], two-stage and three-stage DEA models can be used to evaluate static CEE [25] While the Malmquist model, in conjunction with DEA models, is mostly used to measure dynamic efficiency. Unlike the traditional CCR or BCC models, the SBM model is a non-radial, non-angle model that allows for various proportional changes in input and output variables (including non-angle and slack variables). The SBM model is uniquely suited to measure research components that contain undesirable outputs and is able to take into account the relationship between multiple input and output indicators, resulting in a more comprehensive assessment of efficiency. However, the SBM model suffers from the defect that the efficient DMUs are all equal to one, which precludes comparisons. On the other hand, the opposite is true for the super-efficiency model. Thus, this study supplements the undesirable output SBM model with the undesirable output super-efficiency SBM model.

The formulation of low-carbon strategies for regions or industries must be based on measurements of either the total amount or efficiency of carbon emissions. Index decomposition analysis (IDA), structural decomposition analysis (SDA) and stochastic impacts by regression on population, affluence, and technology (STIRPAT) were used to analyze the factors that determine total carbon emissions [26–28]. The factors that affect CEE was revealed by ordinary least squares (OLS), the spatial Durbin model, and the Tobit regression model [29, 30]. In terms of factor selection, economic growth, industrial structure, energy structure, innovation level, and foreign investment was mostly considered. Since CEE values are truncated data, and ordinary OLS models do not take the efficiency values as a finite dependent variable, so the regression results are biased and inconsistent. The Tobit model is able to handle continuous response variables with upper or lower bounds, which makes it more suitable for studying the factors affecting CEE.

With the increasingly serious problem of climate change, predicting carbon emissions has become a topic of global significance. Most prediction models in early studies mainly relied on statistical methods, such as regression analysis and time series analysis [31, 32]. However, these methods have limitations in dealing with nonlinear and complex causal relationships. To solve this problem, machine learning and artificial intelligence technology have been applied to predict carbon emissions. Zhang proposed a carbon emission prediction model based on deep learning [33], which can effectively handle nonlinear relationships and high-dimensional data. Yin predicted China’s carbon emissions based on a gray prediction model [34], revealing that China’s carbon emissions are closely tied to economic growth and energy consumption, and industrial restructuring is an important means to reduce carbon emissions. Karamouz constructed a system dynamics model to simulate the feedback relationship between the socioeconomic system and the climate system and predicted carbon emissions based on this model [35]. Comparably, system dynamics has the advantage of simulating and analyzing the interactions, feedback mechanisms, nonlinear effects among the factors influencing carbon emissions, and analyze the impacts of initiatives to minimize them.

Jiangsu is an economically developed province in eastern China with an advanced logistics cluster that spans across multiple industries. In 2020, the province’s total social logistics amounted to 32.88 trillion-yuan, accounting for approximately 11% of the national, which shows that the logistics in Jiangsu are at the forefront in China. In such situations, the LCE in Jiangsu remains high, which seriously affects ecological environment and hinders meeting the province’s “carbon peaking and carbon neutrality goals.” Therefore, it is necessary to account and predict the LCE in Jiangsu and formulate adaptive low-carbon strategies for it.

Scholars generally limit the research object to national or provincial areas due to data availability. The level of economic development varies across municipalities, and the implementation of the same development policy may be deviated. Meanwhile, most scholars tend to select the variables affecting CEE from the perspective of the economy, energy, technology, and industry and seldom consider the impact of ecological indicators. Therefore, it is urgent to further study LCE from the following aspects: (ⅰ) analysising of LCE from a municipal perspective; (ⅱ) including ecological indicators in the exploration of LCEE influencing factors; (ⅲ) constructing a system dynamics model of LCE.

This study takes all 13 cities in Jiangsu as the research obiects, mainly to achieve the following goals: (i) integrate the logistics carbon footprint and carbon emission efficiency accounting model; (ii) propose a LCE prediction method based on the system dynamics model; (iii) analyze the influence factors of the efficiency of LCE; (iv) explore the feasible low-carbon strategies for logistics. Therefore, this study first introduces the research progress and development dilemma and investigates the development of logistics and the carbon emissions of study area. Then,constructing the technical framework of LCE composed of three parts: measurement, prediction, and strategy. Next, we conduct an empirical study of Jiangsu. In the discussion, we discuss the drivers of LCE efficiency, the evolution trend of LCE in two scenarios, and formulate low-carbon development strategies for logistics.

## 2. Data and methodology

### 2.1 Case summary

Jiangsu is a developed coastal province in eastern China. In 2022, the province’s GDP reached 12.29 trillion yuan, the second highest in the country. The total value of foreign trade imports and exports was 5.45 trillion yuan. Logistics has supported the growth of Jiangsu’s economy. In 2021, the four modes of transportation (highway, railway, water, and air) completed a total of 3.07 billion tons of freight volume and 1244.17 billion tons/km of freight turnover. The output value of the transportation, warehousing and post and telecommunications(P&T) industry reached 346.62 billion yuan. At the same time, the total energy consumption of Jiangsu was 326.73 million tons of standard coal, and the energy consumption of the transportation, warehousing and post and telecommunications industry was 26.07 million tons of standard coal. Among them, the total energy consumption and carbon emissions of highway and waterway transportation in Jiangsu reached 15.62 million tons of standard coal and 27.67 million tons of carbon dioxide, respectively. Therefore, the total carbon emissions of the Jiangsu are the third highest in the country. The contradiction between the development of logistics and carbon emission constraints in Jiangsu has become increasingly acute, and there is an urgent need for low-carbon strategies.

### 2.2 Technical framework of LCE

**Fig 1:**
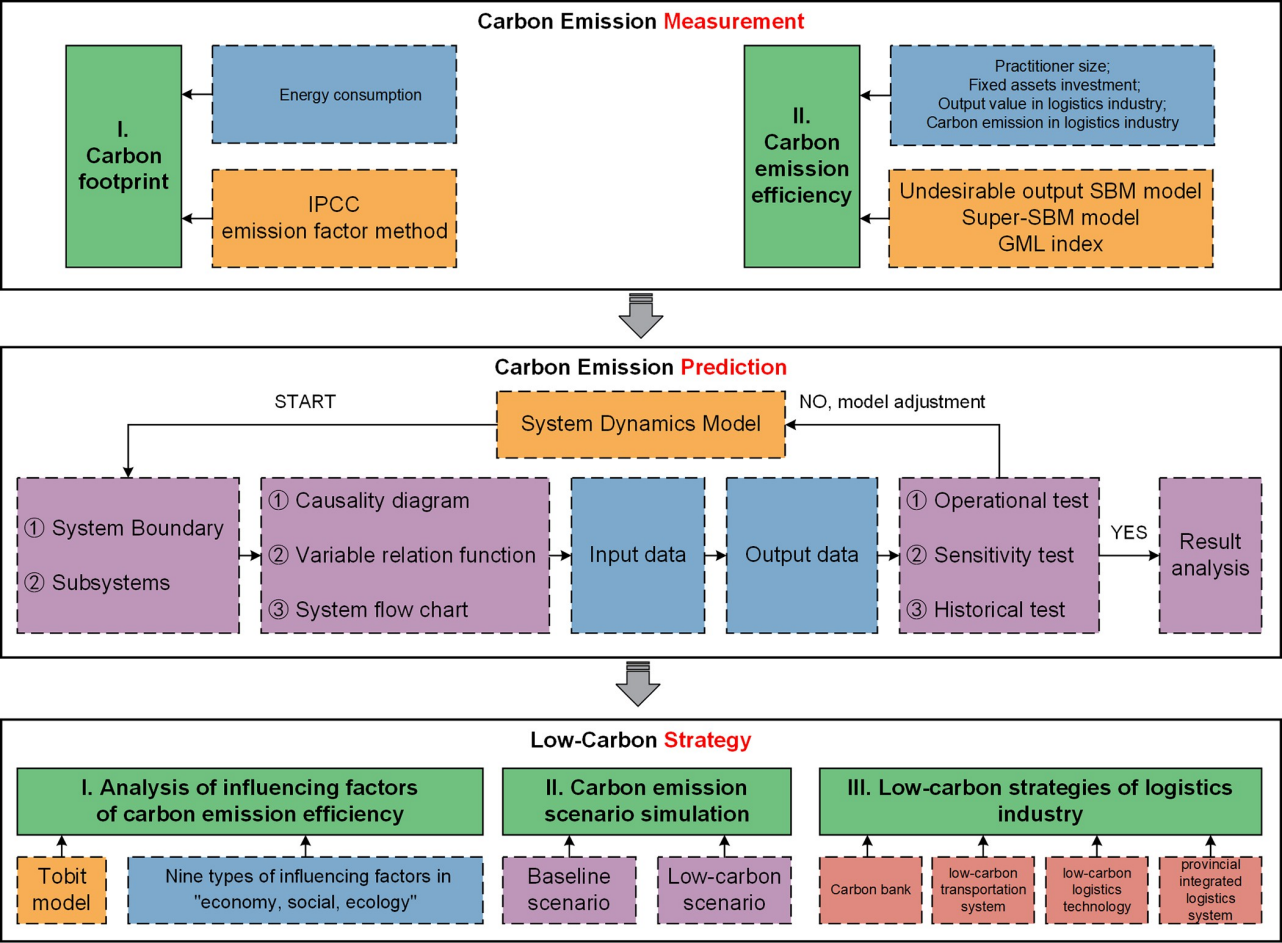
Technical framework of LCE.

The technical framework of logistics carbon emissions constructed in this study consists of three parts: (i) carbon emission measurement; (ii) carbon emission prediction; and (iii) low-carbon strategy (see Fig 1).

#### 2.2.1 Carbon emission measurement

*(1) Carbon footprint accounting model*. This study uses the IPCC emission factor method to measure the carbon footprint of logistics (LCF). The following eight core energy sources are involved in logistics: ***raw coal*, *gasoline*, *kerosene*, *diesel*, *fuel oil*, *liquefied petroleum gas*, *natural gas and electricity***. Using the method proposed by Shan. [36], the provincial energy consumption data were converted to the city scale using GDP. Based on energy consumption data and the conversion coefficient of standard coal and carbon conversion reference coefficient **(see S1 File)**, the LCF is calculated by the following equation:

$$\begin{array}{l} E_{c,i}=E_{p,i}\times \mu =E_{p,i}\times \frac{G_{c}}{G_{p}}\\ LCF=\sum_{i=1}\left(E_{c,i}\times \varepsilon_{i}\times \theta_{i}\right)\times \lambda \times \frac{44}{12} \end{array}$$ (1)

where *E*_*c*,*i*_ and *E*_*p*,*i*_ are the *i*-th total energy consumption at municipal and provincial levels, respectively, 10^4^ tons; *μ* is the ratio of municipal logistics output value (*Gc*) to provincial logistics output value (*G*_*p*_); *ε*_*i*_ is the conversion coefficient of standard coal for the *i*-th energy, kg standard coal/kg; *θ*_*i*_ is the carbon conversion reference coefficient for the *i*-th energy, kg/kg standard coal; *λ* is the correction factor; 44/12 is the conversion factor of carbon atoms to carbon dioxide.

*(2) Carbon emission efficiency accounting model*. To conduct a global comparative analysis of carbon emission efficiency of the logistics (LCEE), this study uses a combination of DEA and super-DEA models for measurement. Because the research samples in the actual logistics generally have non-constant returns to scale, the DEA model based on variable returns to scale (VRS) is chosen. Therefore, this study uses the SBM model to measure the static LCEE. Each DMU contains three elements: (i) input, (ii) desirable output, and (iii)undesirable output [37]. To further analyze these effective DMUs, this study applies the undesirable output SBM model combined with Super-SBM model. The practitioners and the fixed assets investment of logistics are selected as input indicators, the logistics output value as the desirable output indicator, and the LCF as the undesirable output indicator. Then, this study constructs the Global Malmquist-Luenberger(GML) index based on the SBM model and decomposes the index into the technical efficiency change index(TECI) and technical change index(TCI) to analyze dynamic efficiency of LCE [38].

Undesirable output SBM model and Super-SBM model

Suppose there are a total of *N* DMUs, each representing a city (n = 1,2,3…N), and for each DMU_n_, there are *M* input variables, *P* desirable output variables, and *Q* undesirable output variables. Define the input vector *X*, the desirable output vector *Y*, and the undesirable output vector *Z*:

$$X=(x_{n})\in R^{M\times N},Y=(y_{n})\in R^{P\times N},Z=(z_{n})\in R^{Q\times N}$$ (2)

Let *X* > 0, *Y* > 0 and *Z* > 0. Then the production possibility set *C* is

$$\begin{array}{l} C=\left\{(x,y,z)|x_{m}\geq \sum_{n=N}^{N}x_{mn}\lambda_{n},y_{p}\leq \sum_{n=N}^{N}y_{pn}\lambda_{n},z_{p}\geq \sum_{n=N}^{N}z_{qn}\lambda_{n}\right\}\\ s.t.x_{mn}\geq 0,y_{pn}\geq 0,z_{pn}\geq 0,\lambda_{n}\geq 0 \end{array}$$ (3)

where *x*_*mn*_ is the *m*-th input of city n; *y*_*pn*_ is the *p*-th desirable output of city *n*; *z*_*qn*_ is the *q*-th undesirable output of city *n*; and *λ*_*n*_ is the weight of city *n*, indicating the linear combination coefficient of the decision unit on the frontier; The three inequalities in *C* indicate that the actual input level and the actual undesirable output are greater than the frontier level and the actual desirable output level is less than the frontier level, respectively. The DMU (*x*_*0*_, *y*_*0*_, *z*_*0*_) is evaluated using a non-oriented SBM model for undesirable outputs assuming the undesirable outputs is VRS:

$$\begin{array}{l} \rho =\min\frac{1-\frac{1}{M}\sum_{m=1}^{M}\frac{s_{m}^{x}}{x_{m0}}}{1+\frac{1}{P+Q}(\sum_{p=1}^{P}\frac{s_{p}^{y}}{y_{p0}}+\sum_{q=1}^{Q}\frac{s_{q}^{z}}{z_{q0}})}\\ s.t.\;x_{m0}=\sum_{n=1}^{N}x_{mn}\lambda_{n}+s_{m}^{x},\forall m\\ \;y_{p0}=\sum_{n=1}^{N}y_{pn}\lambda_{n}-s_{p}^{y},\forall p\\ \;z_{q0}=\sum_{n=1}^{N}z_{qn}\lambda_{n}+s_{q}^{z},\forall q\\ \;s_{m}^{x}\geq 0,s_{p}^{y}\geq 0,s_{q}^{z}\geq 0,\lambda_{n}\geq 0,\sum_{n=1}^{N}\lambda_{n}=1,\forall m,p,q,n \end{array}$$ (4)

DMU(*x*_*0*_,*y*_*0*_,*z*_*0*_) is evaluated using a non-oriented Super-SBM model with undesirable outputs:

$$\begin{array}{l} \rho =\min\frac{1+\frac{1}{M}\sum_{m=1}^{M}\frac{s_{m}^{x}}{x_{m0}}}{1-\frac{1}{P+Q}(\sum_{p=1}^{P}\frac{s_{p}^{y}}{y_{p0}}+\sum_{q=1}^{Q}\frac{s_{q}^{z}}{z_{q0}})}\\ s.\;t.x_{m0}\geq \sum_{n=1,\neq 0}^{N}x_{mn}\lambda_{n}-s_{m}^{x},\forall m\\ \;y_{p0}\leq \sum_{n=1,\neq 0}^{N}y_{qn}\lambda_{n}+s_{p}^{y},\forall p\\ \;z_{q0}\geq \sum_{n=1,\neq 0}^{N}z_{qn}\lambda_{n}-s_{q}^{z},\forall q\\ \;1-\frac{1}{P+Q}(\sum_{p=1}^{P}\frac{s_{p}^{y}}{y_{p0}}+\sum_{q=1}^{Q}\frac{s_{q}^{z}}{z_{q0}})>0\\ \;s_{m}^{x}\geq 0,s_{p}^{y}\geq 0,s_{q}^{z}\geq 0,\lambda_{n}\geq 0,\sum_{n=1}^{N}\lambda_{n}=1,\forall m,p,q,n \end{array}$$ (5)

where $\frac{1}{M}\sum_{m=1}^{M}\frac{s_{m}^{x}}{x_{m0}}$ is the average value of the input redundancy *M* as a proportion of actual input; $\frac{1}{P+Q}(\sum_{p=1}^{P}\frac{s_{p}^{y}}{y_{p0}}+\sum_{q=1}^{Q}\frac{s_{q}^{z}}{z_{q0}})$ is the average value of the output shortfall of *P+Q* as a proportion of the actual output; $x_{m0},y_{p0},z_{q0}$ are the *m*-th input, the *p*-th desirable output and the *q*-th undesirable output, respectively; $s_{m}^{x},s_{p}^{y},s_{q}^{z}$ are the slack variables for the *m*-th input, the *p*-th desirable output and the *q*-th undesirable output, respectively; *ρ* is a relative value, under the SBM model: when *ρ* = 1, it indicates that the point is above the frontier surface, that the carbon efficiency is effective, and that the city brings more output and less carbon emissions for a given input compared to other cities; Conversely, when *ρ*<1, it indicates that the point is below the frontier surface and the carbon emission efficiency is ineffective or weakly effective; while under Super-SBM: when *ρ* = 1, it indicates that the carbon emission efficiency is ineffective or weakly effective; when *ρ*>1, it indicates that the carbon emission efficiency is effective.

② GML Index

Based on the SBM model, the GML index is further used to analyze the dynamic changes of CEE, which can be expressed as:

GMLcG(xt,yt,zt,xt+1,yt+1,zt+1)=EcG(xt+1,yt+1,zt+1)EcC(xt,yt,zt)ECc=EcT(xt+1,yt+1,zt+1)EcT(xt,yt,zt)TCc=[EcG(xt+1,yt+1,zt+1)EcT(xt+1,yt+1,zt+1)EcG(xt,yt,zt)EcT(xt,yt,zt)] (6)

where $GML_{c}^{G}$ is the change in the GML index of the DMU from year t to t+1; $E_{c}^{G},E_{c}^{T}$ denote the SBM directional distance functions based on the global and current production sets, respectively; *EC*_*c*_ is the technical efficiency change index; When *EC*_*c*_>1, it means that the DMU is closer to the current frontier in period t+1 than in period t, i.e., more efficient; *TC*_*c*_ is the technological change index, when *TC*_*c*_>1, it means that DMU is closer to the global frontier in period t+1 than in period t, i.e., technological progress.

#### 2.2.2 Carbon emission prediction method based on system dynamics model

*(1) System boundary and subsystem*. The system dynamics (SD) model can depict and analyze the interactions, feedback mechanisms and nonlinear effects among the factors influencing carbon emissions, as well as analyze the impacts of various carbon emission control schemes. Confirming the system boundary is the first step in establishing the SD model. In this study, the system boundary of LCE is determined as the whole Jiangsu, the starting point is 2013, the end point is 2030, and the time step is set to one year.

Through ***Vensim***, this study constructs a LCE SD model including an economy subsystem, population subsystem, energy subsystem and environment subsystem. The four subsystems are interrelated, and there is a causal relationship between them. **S2 File** shows the variables contained in the four subsystems.

*(2) System causality diagram*. This study illustrates three causal feedback loops to construct the system causality diagram, **S3 File.**

**Fig 2:**
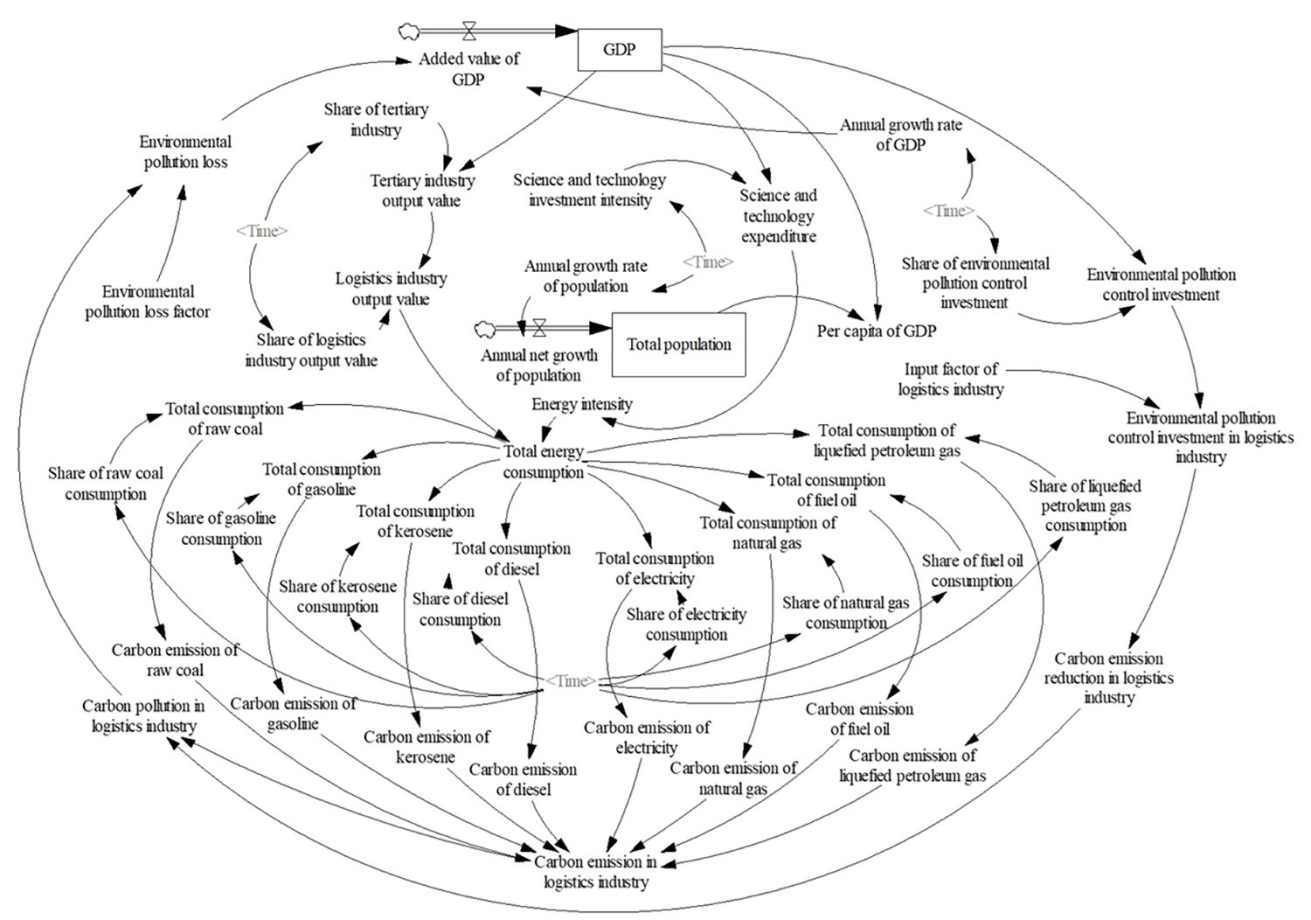
Flow chat of system of LCE.

*(3) Flow chart of the system*. According to the causal diagram of the system, the characters of each variable and the relationship between them, the flow chart of the LCE system is drawn (see Fig 2). The model contains 48 variables, including two horizontal variables, two rate variables, two constants and 42 auxiliary variables. The simulation of the system is divided into two stages. The first stage (2013–2020) is used to test and debug the simulation, and determine the parameters of the system. The second stage (2021–2030) is used to predict LCE. Based on the validity of the model, it is simulated with a step of 1 year.

The parameters of the system variables are set mainly through ratio analysis, direct assignment, literature references and table functions. For space reasons, the main model parameter equations are placed in **S4 File**, taking Suzhou city as an example. The annual GDP growth rate is set as 5.5% for 2021–2025 and 5.0% for 2026–2030 in accordance with the " The Fourteenth Five-Year Plan for National Economic and Social Development of Jiangsu Province and the Outline of Visionary Goals for 2035", while the remaining variables are calculated at the average annual growth rate for 2013–2020.

### 2.3 Data sources

China has not yet defined logistics as an separate industry in its own right. In fact, transportation, warehousing, P&T are the core components of logistics activities. Meanwhile, according to the "China Tertiary Industry Statistical Yearbook", the total added value of these three industries accounts for more than 83% of the total added value of logistics. Therefore, this study selects the relevant statistical data from these three industries to represent logistics in Jiangsu. The detailed description and source of the data are shown in Table 1.

**Table 1:** Data source.

Date	Source	Unit
Total consumption of eight types of energy in Jiangsu	*China Energy Statistics Yearbook (2014–2021)*	10^4^ tons
Gross Domestic Product in Jiangsu	*Jiangsu Statistical Yearbook (2014–2021)*	10^8^ yuan
Gross Domestic Product in each city	*Statistical Yearbook (2014–2021) by City*	
Per capita of GDP in each city		10^8^ yuan /person
Tertiary industry output value in each city		10^8^ yuan
Logistics output value in each city		
Fixed assets investments of logistics in each city		
Science and technology expenditure in each city		
Environmental pollution control investment in each city		
Highway mileage in each city		kilometers
Area in each city		Square kilometers
Total population in each city		person
Urban population in each city		
Size of the logistics practitioners in each city		
Number of college students per 10,000 in each city		

## 3. Results

### 3.1 Carbon emissions measurement of logistics in Jiangsu

#### 3.1.1 LCF in Jiangsu

**Fig 3:**
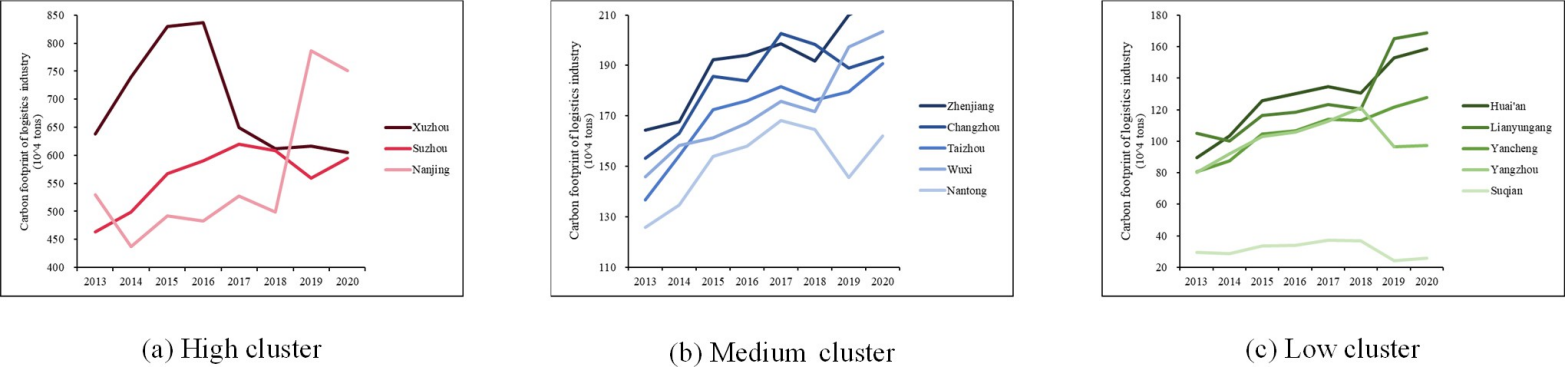
LCF in Jiangsu from 2013 to 2020.

The city-level LCF in Jiangsu from 2013 to 2020 is shown in Fig 3. All 13 cities could be divided into three clusters. The high carbon emission cluster consistently comprises Xuzhou, Suzhou and Nanjing. On average, these three cities account for approximately 40% of the LCF of Jiangsu. The medium carbon emission cluster comprises Zhenjiang, Changzhou, Taizhou, Wuxi and Nantong. The low carbon emissions cluster comprises Yancheng, Huai’an, Lianyungang, Yangzhou and Suqian.

#### 3.1.2 LCEE in Jiangsu

**Fig 4:**
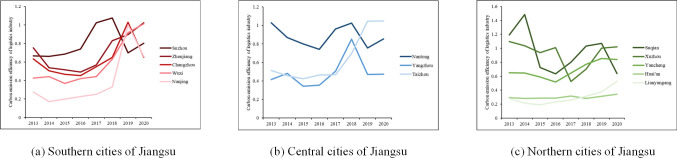
LCEE in Jiangsu from 2013 to 2020.

*(1) Static efficiency*. The static LCEE in Jiangsu from 2013 to 2020 is shown in Fig 4. The average value was 0.63, which was considered moderate. The values of the central region (including Yangzhou, Taizhou, and Nantong) were higher than those of the northern region (including Huai’an, Yancheng, Suqian, Xuzhou, and Lianyungang), and also higher than those of the southern region (including Suzhou, Nanjing, Wuxi, Changzhou, and Zhenjiang). The growth rates of the central, northern, and southern regions were 21.43%, -4.19% and 50.65% respectively. In terms of cities, the static efficiencies of Yangzhou, Huai’an, Yancheng and Lianyungang were not effective, with Huai’an and Lianyungang consistently ranking last. The top three cities in terms of average annual static efficiency are Suqian, Xuzhou and Nantong. Among them, Xuzhou is the city with the most years in which the LCEE has reached an effective level.

Then, we analyze the slack variables of cities with ineffective LCEE. The existence of a slack variable indicates that this indicator is the key point for the improvement of the LCEE of a city, and the value of the slack variable indicates the gap that can be improved. Taking 2020 as an example, see Table 2. For the desirable output, only Suqian has a slack variable, which indicates that the logistics output value in Suqian still has room for growth, while the remaining cities have reached the optimal under the existing conditions. Wuxi, Zhenjiang, Taizhou and Xuzhou’s practitioners and fixed assets investments of logistics had slack variables, indicating that these cities have redundancy in these two aspects. For the undesirable output (LCE), Suzhou, Nanjing, Changzhou, Huai’an and Lianyungang showed slack variables, indicating that there remain carbon emission reductions of logistics to be enhanced in these five cities. Among them, Nanjing, with the largest slack variable value, should have the largest reduction scale of LCE.

**Table 2:** Slack variable values of input-output indicators of logistics in Jiangsu.

	Practitioners of logistics	Fixed assets investments of logistics	Logistics output value	LCE
**Suzhou**	1.21	67.77	0.00	13.78
**Nanjing**	1.34	123.66	0.00	176.48
**Wuxi**	0.00	0.00	0.00	0.00
**Changzhou**	0.00	197.96	0.00	2.25
**Zhenjiang**	0.00	0.00	0.00	0.00
**Yangzhou**	0.79	175.88	0.00	0.00
**Taizhou**	0.00	0.00	0.00	0.00
**Nantong**	0.12	117.62	0.00	0.00
**Huai’an**	0.69	164.72	0.00	61.02
**Yancheng**	0.13	110.93	0.00	0.00
**Suqian**	0.13	141.66	0.29	0.00
**Xuzhou**	0.00	0.00	0.00	0.00
**Lianyungang**	1.27	18.92	0.00	48.29

*(2) Dynamic efficiency*. To study the dynamic change pattern of LCEE and its decomposition terms in 13 cities in Jiangsu, TECI and TCI of each city were calculated by using the GML index, as shown in Tables 3 and 4. The TECIs of Suzhou, Changzhou, Zhenjiang, Nantong, and Suqian were all equal to one in the study period, indicating that the fluctuations in LCEE in these five cities are attributable to the TCI. The TCIs of Suzhou, Changzhou, and Zhenjiang were greater than one in most years, indicating that these cities need to emphasize the operational logistics efficiency. While the TCIs of Nantong and Suqian were smaller than one in most of the years, they need to devote themselves to improving efficiency and technology simultaneously. The TECIs of Yangzhou, Huai’an, Yancheng, Lianyungang were greater than one in most years, and the TCIs were less than one in most years, indicating that these four cities should focus on innovation of logistics technology.

**Table 3:** Technical efficiency change index.

	2014/2013	2015/2014	2016/2015	2017/2016	2018/2017	2019/2018	2020/2019
**Suzhou**	1.00	1.00	1.00	1.00	1.00	1.00	1.00
**Nanjing**	0.61	1.30	1.12	0.85	1.10	2.69	0.82
**Wuxi**	1.27	0.90	1.25	0.90	0.88	1.26	1.00
**Changzhou**	1.00	1.00	1.00	1.00	1.00	1.00	1.00
**Zhenjiang**	1.00	1.00	1.00	1.00	1.00	1.00	1.00
**Yangzhou**	1.33	0.86	1.05	1.05	1.31	0.74	1.15
**Taizhou**	0.97	0.94	1.10	1.00	1.00	1.00	1.00
**Nantong**	1.00	1.00	1.00	1.00	1.00	1.00	1.00
**Huai’an**	0.96	1.48	1.08	1.24	0.78	1.05	1.12
**Yancheng**	1.18	0.99	0.89	1.00	1.07	1.27	1.00
**Suqian**	1.00	1.00	1.00	1.00	1.00	1.00	1.00
**Xuzhou**	1.00	1.00	1.00	0.66	1.52	1.00	1.00
**Lianyungang**	0.73	1.26	1.50	2.14	1.00	0.53	1.87

**Table 4:** Technical change index.

	2014/2013	2015/2014	2016/2015	2017/2016	2018/2017	2019/2018	2020/2019
**Suzhou**	0.99	1.04	1.08	1.35	1.00	0.70	1.15
**Nanjing**	1.03	0.89	1.01	1.31	1.20	1.12	0.81
**Wuxi**	0.82	0.92	0.91	1.17	1.63	1.15	1.09
**Changzhou**	0.80	0.92	0.97	1.21	1.18	1.54	0.65
**Zhenjiang**	0.71	0.96	0.95	1.16	1.46	1.08	1.12
**Yangzhou**	0.87	0.83	0.98	1.35	1.29	0.75	0.87
**Taizhou**	0.91	0.99	1.00	1.01	1.49	1.44	1.00
**Nantong**	0.87	0.92	0.93	1.29	1.04	0.76	1.13
**Huai’an**	1.00	0.70	0.92	0.90	1.14	1.07	0.97
**Yancheng**	0.85	0.92	0.99	1.24	1.11	0.87	0.98
**Suqian**	1.00	0.72	0.88	1.26	1.25	1.00	0.64
**Xuzhou**	1.00	0.94	1.06	0.80	0.88	1.43	1.00
**Lianyungang**	1.03	0.72	0.81	0.52	1.19	2.27	0.74

### 3.2 Carbon emissions prediction of logistics in Jiangsu

#### 3.2.1 Model calibration

Before the prediction, we tested the constructed SD model for operation, sensitivity, and historical results. **See S5 File.**

#### 3.2.2 Analysis of prediction results

The forecast results of LCE in 13 cities of Jiangsu from 2021 to 2030 are shown in Table 5. According to the analysis of the prediction results, 13 cities in Jiangsu can be divided into three categories: (ⅰ) continuously rising cities (Suzhou, Nanjing, Wuxi, Huai’an, Yancheng and Lianyungang), (ii) continuously declining cities (Changzhou, Nantong, Suqian and Xuzhou), and (iii) fluctuating cities (Zhenjiang, Yangzhou and Taizhou). From the perspective of LCE, Nanjing, Suzhou and Xuzhou still rank among the top three in the province. However, since the LCE of Xuzhou has been decreasing year by year since 2021, Nanjing will become the city with the largest LCE in Jiangsu by 2030. The LCEs of Yangzhou and Suqian are consistently ranking last of Jiangsu, and Suqian is the city with the smallest LCE in 2030.

**Table 5:** Predicted results of LCE in Jiangsu (2021–2030).

10^4^ tons	2021	2022	2023	2024	2025	2026	2027	2028	2029	2030
**Suzhou**	595.68	604.81	613.14	622.29	629.62	636.60	643.26	648.33	653.44	658.76
**Nanjing**	699.73	716.37	730.14	743.51	755.45	767.53	779.72	788.79	800.41	810.67
**Wuxi**	205.20	210.07	214.71	219.65	224.34	228.78	232.31	236.31	240.05	244.37
**Changzhou**	184.17	182.19	180.47	178.65	176.89	174.79	172.40	169.75	166.93	164.46
**Zhenjiang**	226.46	228.12	227.00	229.11	236.31	243.07	249.42	255.74	262.02	268.37
**Yangzhou**	94.22	92.41	90.31	87.95	85.64	82.97	82.84	85.26	83.16	80.48
**Taizhou**	174.14	174.65	174.97	175.16	175.26	175.35	175.04	174.59	174.05	173.39
**Nantong**	154.53	151.19	147.86	144.69	141.43	138.43	134.73	131.48	127.97	124.65
**Huai’an**	147.00	152.09	157.50	163.00	168.83	174.78	180.60	186.48	191.98	192.81
**Yancheng**	122.95	126.00	129.10	131.88	134.74	137.27	139.33	141.08	143.74	146.18
**Suqian**	22.75	21.47	20.18	19.14	17.95	16.86	15.77	14.73	13.70	12.78
**Xuzhou**	582.81	555.69	530.16	504.51	480.44	467.59	449.07	431.00	413.63	396.44
**Lianyungang**	147.88	151.72	155.65	159.50	163.23	167.26	170.58	174.24	177.59	181.33

## 4. Discussion

### 4.1 Analysis of the influencing factors of LCEE in Jiangsu

This study analyzes LCEE by selecting nine indicators from three aspects: economy, society and ecology. The specific variable explanations are shown in Table 6. The selection of the first several indicators can be confirmed by previous studies. For example, Sun found that economic level, industrial structure, energy intensity and urbanization rate can affect the CEE of resource-based cities [39]; Sun argued that degree of openness can negatively affect the CEE of Chinese cities [40]. In the current research on CEE, scholars seldom take ecological factors into account, so this study also selected the forestland area as one of the ecological indicators to more comprehensively analyze the factors affecting LCEE.

**Table 6:** Influencing factors of LCEE.

Category	Symbol	Indicator	Explanation of indicator	Unit
**Economy**	**EL**	Economic level	Per capita of GDP	10^8^ yuan
	**IS**	Industry structure	The proportion of logistics output value to tertiary industry output value	%
	**O**	Degree of opening to the outside world	The proportion of total import and export trade to GDP	%
**Society**	**FAU**	Utilization rate of fixed assets	The ratio of logistics output value to fixed asset investment of logistic	-
	**ISL**	Infrastructure level	The ratio of road miles to area	-
	**UR**	Urbanization rate	The proportion of urban population to population	%
	**EP**	Education penetration rate	Number of college students per 10,000 people	person
**Ecology**	**EI**	Energy intensity	energy consumption per logistics output value	tons / 10^4^ yuan
	**ECO**	Ecological environment	The area of forest land	Square kilometers

Further analysis based on indicator selection. For economy: (ⅰ) economic development will inevitably require energy consumption; (ⅱ) industrial structure is one of the key factors that affects CEE, there is no agreement on the positive or negative impact of industrial structure in previous studies, thus this factor is a topic of scholarly debate; (ⅲ) higher degrees of openness to the outside world affects the increase in the level of domestic and foreign freight trade, which leads to an increase in energy consumption. For society: (ⅰ) the fixed assets utilization rate and the infrastructure level in the logistics will increase logistics efficiency, thus affecting the LCEE; (ⅱ) the high urbanization rate and the education penetration rate may, on the one hand, lead to technological advancements, and on the other hand, lead to higher energy consumption. For ecology: (ⅰ) energy consumption is one of the most direct drivers of carbon emissions, and energy intensity, as a measure of energy consumption per unit, becomes the key to LCEE; (ⅱ) forestland is the largest proportion of the land carbon sink, so it is also included as a variable. Synthesizing the above analysis, the nine influencing factors were chosen to analyze LCEE.

Due to the different calculation methods and units of each indicator, the explained variables were normalized using the maximum-minimum standardization to avoid the standard deviation caused by the inconsistent dimension, according to the following Formula (7):

$$x_{it}^{'}=\frac{x_{it}-\min(x)}{\max(x)-\min(x)}$$ (7)

Since the efficiency values of DEA model belong to the restricted explained variables, the panel Tobit regression model with the maximum likelihood method was used to carry out the regression analysis to avoid the dispersion of the efficiency values and the biased situation of parameter estimation, who formula shown below (8):

$$Y_{it}=\{\begin{array}{cc} \beta_{0}+\sum_{t=1}^{T}\beta_{t}x_{it}^{'}+\varepsilon_{it} & Y_{it}>0\\ 0 & Y_{it}\leq 0 \end{array}$$ (8)

Based on the above formulation, the corresponding Tobit regression model was developed below (9):

$$\begin{array}{l} Y_{it}=\beta_{0}+\beta_{1}EL_{it}+\beta_{2}IS_{it}+\beta_{3}O_{it}+\beta_{4}FAU_{it}+\beta_{5}ISL_{it}+\\ \;\beta_{6}UR_{it}+\beta_{7}EP_{it}+\beta_{8}EI_{it}+\beta_{9}ECO_{it}+\varepsilon_{it} \end{array}$$ (9)

where *Y*_*it*_ is an explained variable indicating LCEE in city *i* in year *t*; *β*_*0*_ is a constant term; *x*_*it*_ is the original value of explanatory variable (influencing factor) in city i in year t; $x_{it}^{'}$ is the normalized value of explanatory variable; *β*_*t*_ is the regression coefficient of explanatory variable *x*_*it*_, and *t* is the number of explanatory variables (t = 1,2,3…, T); *ε*_*it*_ is a disturbance term, and *ε*_*i*t_ ~ (0, σ^2^).

**Fig 5:**
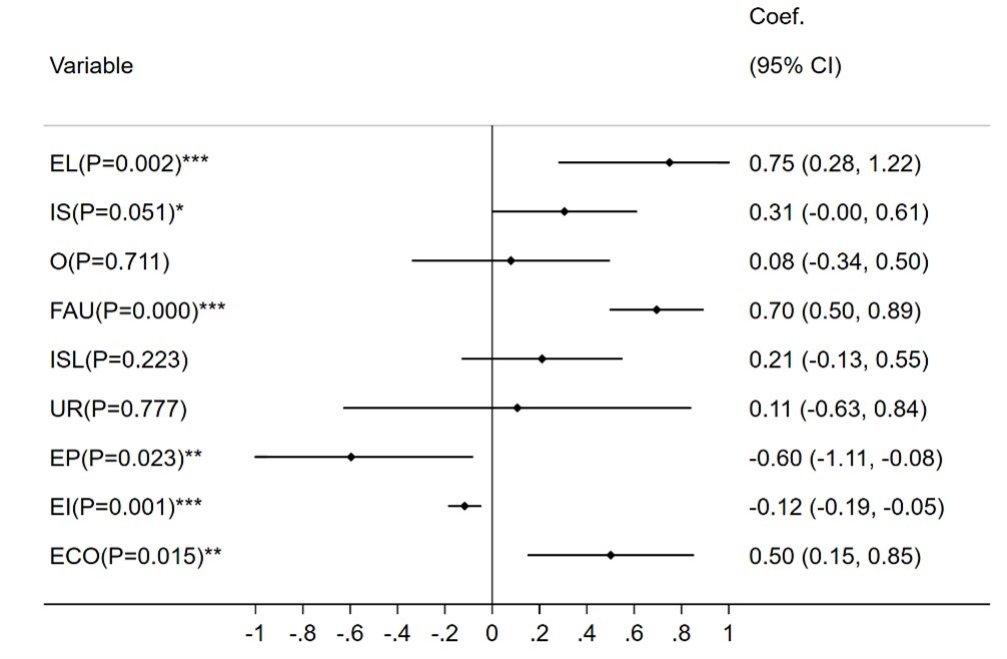
Regression results by Tobit model.

With the help of Stata software, the original data of the selected indicators were subjected to maximum-minimum normalization, and according to the LR test results, strongly rejecting “*H*_0_:*σ*_*u*_ = 0”, the random-effects panel Tobit regression model should be selected for estimation, and the regression results are shown in Fig 5 with the left imputation point at zero and the right imputation point at infinity:

The economic level (R = 0.750), fixed asset utilization rate (R = 0.695) and energy intensity (R = -0.116) are significantly correlated with LCEE at a 1% confidence level. This confirms that a developed economy and high fixed asset utilization rate have a positive effect on improving LCEE, but a low energy utilization rate has an inhibitory effect on LCEE.

The ecological environment and education popularization are significantly correlated with LCEE at the confidence level of 5%. Among them, the ecological environment is positively correlated (R = 0.501). This enlightens us that the improvement of ecosystem quality, including the expansion of forestland area, will promote the absorption of CO_2_, thereby alleviating the pressure on LCE control. Education popularization is negatively correlated (R = -0.596). This implies that the educated population has not resulted in sufficiently skilled labor force to the logistics in Jiangsu to improve LCEE.

### 4.2 Analysis of the carbon emission evolution trend of logistics in Jiangsu based on scenario simulation

In this study combined the SD model with the scenario analysis, then a total of two scenarios, baseline and low-carbon, are set to analyze the future LCE simulations. The use of forecasting in combination with scenario simulation is now widely used in various industry sectors, e.g., Huo used SD and scenario analysis to set up a baseline and a low-carbon scenario to simulate the carbon emissions of commercial buildings in China until 2060 [41]; Similarly, Xiao used the STIRPAT with scenario analysis to study carbon emissions during the operation of a university building in Jiangxi, and set three scenarios of baseline, low carbon, and ultra-low carbon for analysis [42]. However, most scholars only choose a research object for prediction. Different from other studies, the logistics of 13 cities in Jiangsu were chosen by this study, then use the SD model to simulate the LCE of 13 cities in Jiangsu in two scenarios one by one.

The key of scenario simulation lies in the regulatory parameters. This study analyzed in term of economy, energy, and environment were based on the LCE system flow chart. For economy, the annual GDP growth rate was chosen since GDP is the most representative economic indicator. For energy, optimizing the energy structure can effectively reduce LCE, thus the proportion of each energy was chosen. For environment, in addition to LCE, another important indicator in the system flow diagram is logistics carbon emission reduction (LCER), and by backward reasoning of this variable, environmental pollution control investment was included.

The purpose of this study is to carry out LCER actions. LCE and LCER are the two core indicators of the system flow chart and they can best reflect the differences between the two scenarios. The former is an indicator representing the current status of LCE, while the latter is a quantitative indicator representing the measures taken by the logistics to reduce carbon emissions. Therefore, LCE and LCER are chosen as the two indicators for analyzing the evolutionary characteristics:

ⅰ. In this scenario, logistics continues to maintain the current development trend based on the corresponding policies in Jiangsu, which is an annual GDP growth rate that is consistent with the "The Fourteenth Five-Year Plan for National Economic and Social Development of Jiangsu Province and the Outline of Visionary Goals for 2035". Both the energy structure and the Share of environmental pollution control investment have evolved at historical average annual growth rates. This is the scenario explored in the previous Section 3.2.

ii. Low-carbon scenario. In this scenario, logistics tended toward ecological protection, which slows GDP growth, increases the proportion of clean energy use, and increases environmental pollution investment. The specific setting of the scenario parameters is shown in Table 7.

**Table 7:** Scenario parameters.

Scenario	Indicators		
	Annual growth rate of GDP	Energy Structure	Share of environmental pollution control investment
**Baseline scenario**	5.5% growth rate in 2021–2025 and 5.0% growth rate in 2026–2030	According to the historical average annual growth rate	According to the historical average annual growth rate
**Low Carbon Scenario**	4.5% growth rate in 2021–2025 and 4.0% growth rate in 2026–2030	Compared to the baseline scenario, the share of gasoline and diesel decreased by 5%, the share of kerosene decreased by 2%, the share of fuel oil decreased by 1%, and the share of natural gas increased by 13%	Compared to the baseline scenario, the share of environmental pollution investment increased by 0.2%

The LCEs under the low-carbon scenario from 2021–2030 are shown in Table 8. Compared with the baseline scenario, it decreased by an average of 13.28%, with the largest average decrease in Yancheng (13.79%). Table 9 is the comparative data of LCER in 13 cities of Jiangsu under the two scenarios. Under the low-carbon scenario, the LCER of Jiangsu increased by 207.96% on average compared with the baseline scenario. Among them, the least average increase was seen in Suzhou (106.72%) and the largest increase was seen in Taizhou (433%).

**Table 8:** LCE under low-carbon scenario (2021–2030).

10^4^ tons	2021	2022	2023	2024	2025	2026	2027	2028	2029	2030
**Suzhou**	528.33	533.23	531.49	542.77	546.53	550.11	553.49	555.60	557.87	560.44
**Nanjing**	620.61	631.72	633.49	649.41	656.91	664.63	672.50	677.79	685.33	691.79
**Wuxi**	182.00	185.22	186.13	191.59	194.74	197.70	199.89	202.52	204.95	207.90
**Changzhou**	163.35	160.70	156.57	156.01	153.77	151.31	148.64	145.80	142.87	140.29
**Zhenjiang**	200.86	202.82	200.19	202.19	204.72	209.56	214.04	218.51	222.98	227.51
**Yangzhou**	83.56	80.71	76.87	75.85	78.15	77.27	74.37	71.58	68.73	66.06
**Taizhou**	154.45	154.07	151.84	153.02	152.43	151.87	151.01	150.06	149.07	148.03
**Nantong**	137.06	133.40	128.35	126.44	123.06	119.96	116.30	113.08	109.68	106.49
**Huai’an**	130.37	134.58	137.50	143.63	148.47	153.40	155.80	155.02	154.31	169.24
**Yancheng**	109.04	110.64	111.02	113.75	115.78	117.62	119.34	120.89	122.70	124.34
**Suqian**	20.17	18.95	17.52	16.73	15.63	14.62	13.62	12.68	11.75	10.93
**Xuzhou**	516.91	491.01	461.62	443.01	419.98	401.22	386.14	369.43	353.21	337.34
**Lianyungang**	131.16	133.86	135.10	139.38	142.02	144.95	147.25	149.86	152.22	154.93

**Table 9:** The carbon emission reduction of logistics under two scenarios (2021–2030).

Baseline scenario/10^4^ tons	2021	2022	2023	2024	2025	2026	2027	2028	2029	2030
**Suzhou**	3.95	4.61	5.32	6.12	7.00	8.00	9.09	10.31	11.65	13.16
**Nanjing**	3.94	4.11	4.34	4.62	4.94	5.28	5.63	6.00	6.37	6.76
**Wuxi**	0.97	0.96	0.96	0.96	0.97	0.98	0.98	0.98	0.98	0.97
**Changzhou**	0.52	0.52	0.52	0.52	0.53	0.53	0.53	0.53	0.53	0.53
**Zhenjiang**	0.36	0.37	0.42	0.49	0.58	0.69	0.82	0.98	1.16	1.38
**Yangzhou**	0.36	0.41	0.53	0.76	1.12	1.68	2.53	3.81	5.74	8.63
**Taizhou**	0.26	0.23	0.22	0.20	0.19	0.18	0.17	0.16	0.15	0.15
**Nantong**	0.34	0.34	0.34	0.35	0.36	0.37	0.38	0.39	0.40	0.41
**Huai’an**	0.09	0.09	0.09	0.09	0.10	0.11	0.11	0.12	0.13	0.14
**Yancheng**	0.29	0.25	0.23	0.22	0.21	0.20	0.20	0.19	0.18	0.18
**Suqian**	0.29	0.29	0.29	0.28	0.28	0.28	0.28	0.28	0.28	0.28
**Xuzhou**	1.90	1.67	1.56	1.52	1.52	1.54	1.55	1.57	1.59	1.60
**Lianyungang**	0.31	0.30	0.29	0.28	0.27	0.26	0.25	0.24	0.22	0.21
**Low Carbon Scenario/10**^**4**^ **tons**	**2021**	**2022**	**2023**	**2024**	**2025**	**2026**	**2027**	**2028**	**2029**	**2030**
**Suzhou**	6.30	9.53	11.88	13.83	15.63	17.44	19.27	21.19	23.24	25.47
**Nanjing**	6.45	8.78	10.29	11.40	12.32	13.16	13.93	14.67	15.41	16.16
**Wuxi**	1.57	2.02	2.28	2.44	2.54	2.62	2.67	2.71	2.75	2.78
**Changzhou**	0.96	1.36	1.59	1.72	1.81	1.88	1.92	1.96	1.99	2.03
**Zhenjiang**	0.72	0.91	1.04	1.19	1.36	1.55	1.76	2.00	2.27	2.58
**Yangzhou**	0.64	0.91	1.22	1.64	2.21	3.01	4.13	5.70	7.95	11.18
**Taizhou**	0.57	0.81	0.94	1.00	1.04	1.06	1.07	1.07	1.08	1.08
**Nantong**	0.78	1.22	1.48	1.65	1.77	1.86	1.94	2.01	2.08	2.14
**Huai’an**	0.17	0.25	0.30	0.33	0.36	0.39	0.41	0.44	0.46	0.48
**Yancheng**	0.58	0.72	0.80	0.84	0.87	0.89	0.90	0.91	0.91	0.92
**Suqian**	0.45	0.56	0.62	0.66	0.68	0.70	0.71	0.71	0.72	0.72
**Xuzhou**	3.70	4.56	5.07	5.42	5.70	5.93	6.12	6.28	6.44	6.59
**Lianyungang**	0.50	0.65	0.73	0.76	0.77	0.77	0.77	0.76	0.75	0.74

Comparing LCE and LCER under different scenarios in different cities, the comparison figures were shown in **S6 File**. The direct factor affecting LCE lies in the energy consumption and energy structure in the logistics, and the energy consumption is determined by the energy intensity and the logistics output value. The energy intensity and the logistics output value vary from city to city, resulting in LCE in different cities under the low-carbon scenario has experienced different development trends. LCER can be traced back to environmental pollution control investment. The share of environmental control investment in the 13 cities is different, leading to different trends in LCER in each city under the low-carbon scenario.

**Fig 6:**
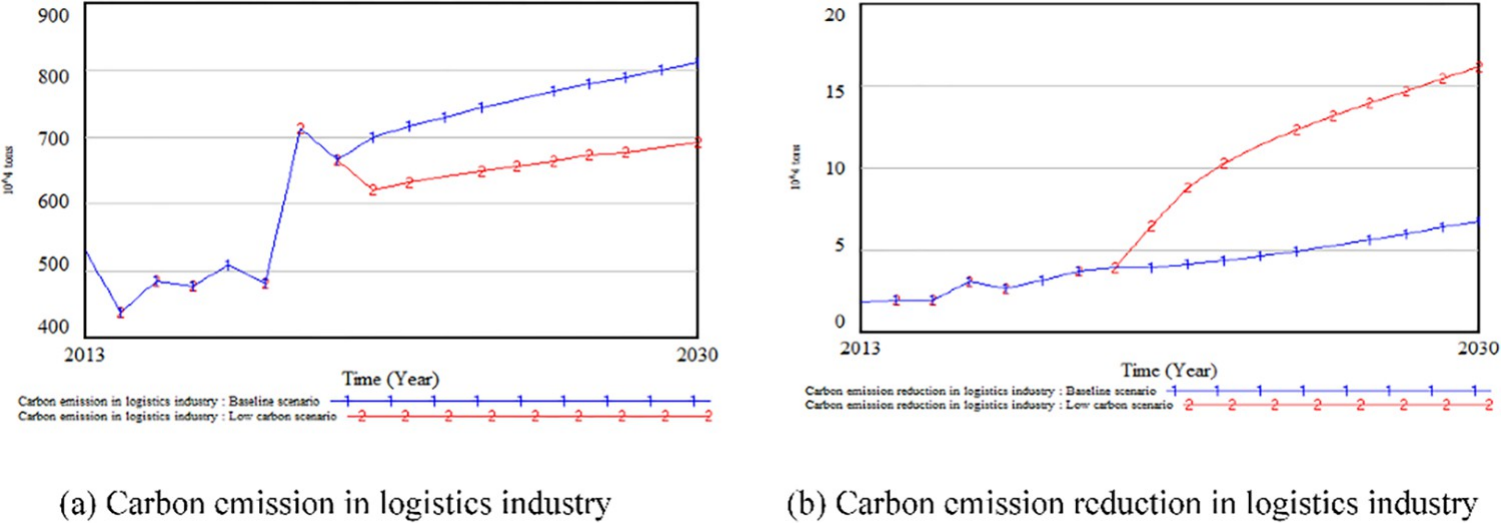
Comparison of trends in the two scenarios (Nanjing).

The LCE of Nanjing continues to rise under the baseline scenario, and reaches the top of the province in 2030. Therefore, Nanjing is used as an example to conduct a comparative analysis at the city scale. Fig 6A shows the LCE between two scenarios. Under the baseline scenario, the average annual growth rate of LCE in Nanjing is 1.65%, and the growth rate from 2030 to 2021 is 15.86%. Under the low-carbon scenario, the average annual growth rate of LCE is 1.22%, and the growth rate from 2030 to 2021 is 11.47%. After 2021, the LCE under the low-carbon scenario will be reduced by 1 million tons compared with the baseline scenario, and the growth rate will be smaller. Fig 6B shows the LCER between two scenarios. Under the low-carbon scenario, the LCER in Nanjing increased by an average of 7.06×10^4^ tons compared with the baseline scenario. The average annual growth rate of the carbon emission reduction increased from 6.18% to 11.12%, and the growth trend was steeper. The above results show that the annual growth rate of GDP, energy structure and proportion of investment in environmental pollution control have a positive effect on energy conservation and emission reduction, and the low-carbon scenario set in this study is conducive to the reduction of LCE in Jiangsu.

### 4.3 Low-carbon strategy of the logistics in Jiangsu

#### 4.3.1 Build a city “carbon bank”

The results show that improving the eco-environment positively contributes to LCEE. The delay of the carbon sink construction is one of the important reasons behind the enormous carbon emissions of Jiangsu. Actually, Jiangsu, with its diversified ecological elements, has certain advantages in the construction of city “carbon bank”. Increasing the establishment of city “carbon banks” will help to improve the ecological environment and enhance the attractiveness of cities, thereby attracting more talent and investment. Therefore, Jiangsu should leverage the diversified ecological factors to build a city “carbon bank”. First, the benefits of carbon sequestration should be incorporated as the standard of city eco-space planning. It is suggested to coordinate the optimal carbon sequestration combination of forestland, cultivated land, wetlands, and ocean. Second, native species of Jiangsu, such as purple heather, red cedar, and ginkgo biloba, should be planted to achieve carbon reduction and economic effects simultaneously. Third, the city carbon bank needs to be equipped with a carbon sink monitoring mechanism to track its evolution in real time.

#### 4.3.2 Construct a low-carbon transportation system

During the study period (2013–2020), there was a redundancy in the fixed asset investment of the logistics in majority cities in Jiangsu. Meanwhile, the fixed asset utilization rate was significantly positively correlated with the LCEE. This indicated that the utilization efficiency of fixed assets of the logistics in Jiangsu was far from the efficient status. Jiangsu should promote the construction of low-carbon transportation system and reduce energy consumption throughout the life cycle of transportation infrastructure. On the one hand, it can help to address the global climate change, and on the other hand, it can improve transportation efficiency and urban spatial efficiency, as well as promote the new energy vehicle industry and facilitate economic transformation and sustainable development. For freight transportation, Jiangsu may accelerate the construction of a low-carbon multimodal transport system and promote “road-to-rail” and“road-to-water” in bulk cargo and long-distance transport. For public transportation, Jiangsu could replace conventional fuel vehicles with new energy vehicles to curb exhaust emissions effectively. For individual travel, Jiangsu should popularize low-carbon shared travel modes such as shared bicycles or electric vehicles, and optimize urban slow-moving systems such as non-motorized lanes.

#### 4.3.3 Cultivate logistics talent and innovate low-carbon logistics technology

The slack variables of the logistics practitioners calculated in this study have redundancy, indicating that the technical ability of the logistics practitioners in Jiangsu is not saturated. At the same time, the TCI of the Jiangsu is mostly less than one. Therefore, Jiangsu must vigorously cultivate professional modern logistics talent and innovate low-carbon logistics technology. The former may improve the quality and efficiency of logistics services; the latter could accelerate the green development of the logistics and promote the transformation of traditional logistics. As the main generator of logistics professionals, universities could improve training modes by teaching basic theoretical knowledge related to low-carbon logistics and training in operating skills of green logistics facilities and equipment. Meanwhile, the government can guide logistics enterprises to expand the recruitment scale of graduate major in low-carbon logistics by preferential policies. Logistics enterprises could integrate artificial intelligence, blockchain, cloud computing, big data, 5G to build a regional cooperation platform to foster information exchange and collaboration between supply chain entities.

#### 4.3.4 Establish a provincial integrated logistics system

There is marked spatio-temporal heterogeneity in the level of the logistics and LCF in all 13 cities of Jiangsu. Provincial integrated logistics systems are suitable for vast and prosperous economies area, it is conducive to the optimization and reallocation of resources, the formation of industrial agglomeration effects, the improvement of provincial logistics operation efficiency, and the cooperation of inter-provincial trade. Therefore, Jiangsu could adhere to establish an integrated provincial logistics system. As a closely linked and highly integrated spatial geographical unit, the metropolitan circle is a prerequisite for developing an integrated provincial logistics system. Each city should focus on leveraging its respective advantages, and serve as a logistics hub according to its own logistics function, driving the synergistic operation of the provincial logistics system.

#### 4.3.5 Implement differentiated low-carbon logistics policies

All 13 cities in Jiangsu can be categorized into high-carbon, medium-carbon, and low-carbon cities based on the level of LCF in this study. The implementation of differentiated low-carbon logistics policies could better fulfill the needs of different regions and logistics enterprises, and improve the operability and flexibility of policy. First, high-carbon cities are encouraged to adopt energy-efficient logistics technologies and increase the utilization of renewable energy by improving the efficiency of power generation equipment and developing energy storage technologies. Second, medium-carbon cities can gradually implement the decarbonization of logistics while expanding the scale of the logistics. Specifically, they should gradually adopt clean energy instead of traditional energy to reduce their dependence on high-carbon energy. At the same time, this type of city can make up the space for logistics development in order to balance the development of regional logistics. Finally, low-carbon cities should fully utilize their remaining carbon capacity. On the one hand, it may continue to maintain and increase the carbon sink. On the other hand, it could undertake more logistics business to share the logistics pressure of medium- and high-carbon cities, to achieve the ultimate goal of reducing the total amount of LCE in the province.

### 4.4 Limitations

When calculating LCF, we cannot directly obtain the energy consumption data of each city limited by the current data statistics and publication principles. Therefore, this study constructs a scale conversion index based on GDP and the logistics output values, which may lead to a gap between the actual performance and calculation results in city scale studies. Then, it is urgent to explore a more persuasive and operable method of transforming carbon emissions from large scale to small or medium scale for a specific industry.

## 5. Conclusions

This study constructs a technical framework of LCE, and takes Jiangsu, China as an example for empirical research: (ⅰ) results found that LCFs in Jiangsu are generally at a high level. In particular, LCFs in Suzhou, Xuzhou, and Nanjing are severe; (ⅱ) LCEE is at a medium level, the efficiency values of the central region were higher than those of the northern and the southern; (ⅲ) the economic level, fixed asset utilization rate, energy intensity, ecological environment, and education popularization are significantly correlated with LCEE; (ⅳ) the low-carbon scenario is helpful in alleviating the logistics carbon emission pressure of Jiangsu. From the perspectives of carbon sink, transportation systems, professional training, low-carbon technological innovation and integration of regional logistics, the following countermeasures are provided for the low-carbon transformation of logistics in Jiangsu: (ⅰ) build a city “carbon bank”; (ⅱ) construct a low-carbon transportation system; (ⅲ) cultivate logistics talent and innovate low-carbon logistics technology; (ⅳ) establish a provincial integrated logistics system; (ⅴ) implement differentiated low-carbon logistics policies. It is expected to provide valuable references for the improvement of logistics in Jiangsu, China.

## Supporting information

S1 File Conversion coefficient of standard coal and carbon conversion reference coefficient for each energy. (DOCX)

S2 File Description of subsystems and main variables. (DOCX)

S3 File System causality diagram. (DOCX)

S4 File The main model parameter equations. (DOCX)

S5 File Model calibration. (DOCX)

S6 File Comparison of trends between the two scenarios in 13 cities in Jiangsu. (DOCX)
