# Supplementary material for: Evaluation and prediction of carbon emission from logistics at city scale for low-carbon development strategy
Source: PLoS One. 2024 Feb 29;19(2):e0298206. doi: 10.1371/journal.pone.0298206 (PMC10903878; doi:10.1371/journal.pone.0298206)
Supplement: S1 File — (DOCX) [file pone.0298206.s001.docx]

**Supplementary Materials**

1. **Conversion coefficient of standard coal and Carbon conversion reference coefficient for each energy**

Table S1. Conversion coefficient of standard coal and Carbon conversion reference coefficient for each energy.

| Coefficient Type (kg ce/kg) | Raw coal | Gasoline | Kerosene | Diesel |
| --- | --- | --- | --- | --- |
| Conversion coefficient of standard coal | 0.7143 | 1.4714 | 1.4714 | 1.4517 |
| Carbon conversion reference coefficient | 1.9003 | 2.9251 | 3.0179 | 3.0959 |
| Coefficient Type (kg ce/kg) | Fuel oil | Liquefied petroleum gas | Natural gas | Electricity |
| Conversion coefficient of standard coal | 1.4286 | 1.7143 | 1.3300 | 0.1229 |
| Carbon conversion reference coefficient | 3.1705 | 3.1013 | 0.4226 | 0.7140 |

Data source: China Energy Statistics Yearbook and General Rules for Calculating Comprehensive Energy Consumption
