# Supplementary material for: Evaluation and prediction of carbon emission from logistics at city scale for low-carbon development strategy
Source: PLoS One. 2024 Feb 29;19(2):e0298206. doi: 10.1371/journal.pone.0298206 (PMC10903878; doi:10.1371/journal.pone.0298206)
Supplement: S2 File — (DOCX) [file pone.0298206.s002.docx]

**Supplementary Materials**

**Description of subsystems and main variables**

Table S2. Description of subsystems and main variables.

| Subsystem | Variable | Variable description | Unit |
| --- | --- | --- | --- |
| Economy subsystem | Gross Domestic Product | The market value of all final goods (products and services) produced by a country or region using factors of production in a given period, GDP | 10^8^ yuan |
|  | Added value of GDP | Added value of GDP in current year | 10^8^ yuan |
|  | Annual growth rate of GDP | Natural growth rate of GDP | % |
|  | Tertiary industry output value | GDP of tertiary industries such as services and tourism | 10^8^ yuan |
|  | Share of tertiary industry | Tertiary industry output value as a percentage of GDP | % |
|  | Logistics output value | Value generated through logistics activities | 10^8^ yuan |
|  | Share of logistics output value | Logistics industry output value as a percentage of tertiary industry output value | % |
|  | Science and technology expenditure | Expenditure by the government and its related departments to support science and technology activities | 10^8^ yuan |
|  | Science and technology investment intensity | Science and technology expenditure as a percentage of GDP | % |
|  | Environmental pollution control investment | Expenditure by the government and its related departments to protect resources and control environmental pollution | 10^8^ yuan |
|  | Share of environmental pollution control investment | Environmental pollution control investment as a percentage of GDP | % |
|  | Environmental pollution control investment in logistics | Investment in environmental pollution management for the logistics industry | 10^8^ yuan |
|  | Input factor of logistics | The proportion of investment in environmental pollution control in logistics industry to the total investment in environmental pollution control | - |
|  | Environmental pollution loss | Losses caused by environmental pollution | 10^8^ yuan |
|  | Environmental pollution loss factor | Conversion coefficient of carbon pollution volume in logistics industry into environmental pollution loss | - |
| Population subsystem | Total population | Year-end resident population of a region | person |
|  | Annual net growth of population | Net increasement of population in current year | person |
|  | Annual growth rate of population | Natural growth rate of population growth | % |
|  | Per capita of GDP | Per capita of Gross Domestic Product | 10^8^ yuan /person |
| Energy subsystem | Total energy consumption | Total consumption of eight types of energy of in logistics industry (converted into standard coal) | 10^4^ tons |
|  | Energy intensity | The amount of energy consumed by 10,000 yuan of logistics industry output value | tons / 10^4^ yuan |
|  | Carbon emission of each energy | Carbon emissions of each energy in logistics industry | 10^4^ tons |
|  | Each energy consumption | Each energy consumption in logistics industry (converted into standard coal) | 10^4^ tons |
|  | Share of each energy consumption | Proportion of each energy consumption to total energy consumption | % |
| Environment subsystem | Carbon emission of logistics | Potential carbon emissions of eight types of energy in logistics industry | 10^4^ tons |
|  | Carbon emission reduction of logistics | Amount of carbon reduced through environmental measures | 10^4^ tons |
|  | Carbon pollution of logistics | The actual amount of carbon emitted into the environment in logistics industry, is the difference between potential carbon emissions and carbon emission reduction | 10^4^ tons |
