# Supplementary material for: Evaluation and prediction of carbon emission from logistics at city scale for low-carbon development strategy
Source: PLoS One. 2024 Feb 29;19(2):e0298206. doi: 10.1371/journal.pone.0298206 (PMC10903878; doi:10.1371/journal.pone.0298206)
Supplement: S3 File — (DOCX) [file pone.0298206.s003.docx]

**Supplementary Materials**

**3. System causality diagram**

Fig S1 shows causality diagram of carbon emission system in logistics, with the following main causal feedback loops:





Note: → indicates that the arrow tail variable affects the arrow variable; + indicates positive feedback; - indicates negative feedback

Fig S1. Causality diagram of system of LCE.

(ⅰ) Gross Domestic Product (GDP) →+ Tertiary industry output value →+ Logistics industry output value →+ Total energy consumption →+ Each energy consumption →+ Carbon emission in logistics industry →+ Carbon pollution in logistics industry →+ Environmental pollution loss →- Gross Domestic Product (GDP). This is a negative feedback loop. The increase of GDP will drive the increase of Tertiary industry output value, which in turn will drive the increase of Logistics industry output value, and the increase of the output value will drive the increase of the total energy consumption in logistics industry, which will increase the carbon emission in logistics industry and cause the negative impact of the environmental pollution loss on the GDP.

(ⅱ) Gross Domestic Product (GDP) →+ Science and technology expenditure →- Energy intensity →+ Total energy consumption →+ Each energy consumption →+ Carbon emission in logistics industry →+ Carbon pollution in logistics industry →+ Environmental pollution loss →- Gross Domestic Product (GDP). This is a positive feedback loop. Growth in the national economy increases expenditures on science and technology, technological expenditure increases the level of technology, which reduces energy intensity, and an increase in energy intensity increases total energy consumption.

(ⅲ) Gross Domestic Product (GDP) →+ Environmental pollution control investment →+ Environmental pollution control investment in logistics industry →+ Carbon emission reduction in logistics industry →- Carbon emission in logistics industry →+ Carbon pollution in logistics industry →+ Environmental pollution loss →- Gross Domestic Product (GDP). This is a positive feedback loop. As the national economy grows, the government will reduce carbon emissions by increasing investment of environmental pollution control, which will in turn increase investment of environmental pollution control in logistics industry, further increasing carbon emission reduction in the logistics industry.
