# Supplementary material for: Evaluation and prediction of carbon emission from logistics at city scale for low-carbon development strategy
Source: PLoS One. 2024 Feb 29;19(2):e0298206. doi: 10.1371/journal.pone.0298206 (PMC10903878; doi:10.1371/journal.pone.0298206)
Supplement: S4 File — (DOCX) [file pone.0298206.s004.docx]

**Supplementary Materials**

**4.** **The main model parameter equations are as follows (taking Suzhou city as an example):**

**GDP** = INTEG (Added value of GDP, Initial value of GDP) Unit: 10^8^ yuan

**Total population** = INTEG (Annual net growth of population, Initial population number) Unit: person

**Added value of GDP** = GDP * Annual growth rate of GDP - Environmental pollution loss Unit: 10^8^ yuan

**Annual net growth of population** = Total population * Annual growth rate of population Unit: person

**Tertiary industry output value** = GDP * Share of tertiary industry Unit: 10^8^ yuan

**Logistics industry output value** = Tertiary industry output value * Share of logistics industry output value Unit: 10^8^ yuan

**Per capita of GDP** = GDP / Total population Unit: 10^8^ yuan

**Science and technology expenditure** = GDP * Science and technology investment intensity Unit: 10^8^ yuan

**Environmental pollution control investment** = GDP * Share of environmental pollution control investment Unit: 10^8^ yuan

**Environmental pollution control investment in logistics industry** = Environmental pollution control investment * Input factor of logistics industry Unit: 10^8^ yuan

**Carbon emission reduction in logistics industry** = DELAY1(Environmental pollution control investment in logistics industry, 2) * Environmental pollution control investment in logistics industry Unit: 10^4^ tons

**Carbon emission in logistics industry** = (Carbon emission of raw coal + Carbon emission of gasoline + Carbon emission of kerosene + Carbon emission of diesel + Carbon emission of fuel oil + Carbon emission of liquefied petroleum gas + Carbon emission of natural gas + Carbon emission of electricity) * 44/12 * correction factor λ Unit: 10^4^ tons

**Carbon pollution in logistics industry** = Carbon emission in logistics industry - Carbon emission reduction in logistics industry Unit: 10^4^ tons

**Environmental pollution loss** = (Carbon pollution in logistics industry * Environmental pollution loss coefficient) / 10000 Unit: 10^8^ yuan

**Total energy consumption** = Logistics industry output value * Energy intensity Unit: 10^4^ tons

**Total consumption of raw coal** = Total energy consumption * Share of raw coal consumption Unit: 10^4^ tons

**Carbon emission of raw coal** = Total consumption of raw coal * Reference coefficient of carburization (raw coal) Unit: 10^4^ tons

**Annual growth rate of GDP** = WITH LOOKUP (time, ([(2013,0)-(2030,1)],(2013,0.06088),(2014,0.0548),(2015,0.067),(2016,0.09962),(2017,0.07379),(2018,0.03432),(2019,0.04859),(2020,0.12632),(2021,0.055),(2022,0.055),(2023,0.055),(2024,0.055),(2025,0.055),(2026,0.05),(2027,0.05),(2028,0.05),(2029,0.05)) Unit: %

**Annual growth rate of population** = WITH LOOKUP (time, ([(2013,0)-(2030,1)],(2013,0.01107),(2014,0.00898),(2015,0.01677),(2016,0.01898),(2017,0.01806),(2018,0.02708),(2019,0.03007),(2020,0.02388),(2021,0.01872),(2022,0.01872),(2023,0.01872),(2024,0.01872),(2025,0.01872),(2026,0.01872),(2027,0.01872),(2028,0.01872),(2029,0.01872)) Unit: %

**Share of tertiary industry** = WITH LOOKUP (time, ([(2013,0)-(2030,1)],(2013,0.464),(2014,0.484),(2015,0.484),(2016,0.504),(2017,0.503),(2018,0.5),(2019,0.516),(2020,0.525),(2021,0.5779),(2022,0.5883),(2023,0.5988),(2024,0.6096),(2025,0.6206),(2026,0.6317),(2027,0.6431),(2028,0.6547),(2029,0.6665),(2030,0.6784)) Unit: %

**Share of logistics industry output value** = WITH LOOKUP (time, ([(2013,0)-(2030,1)],(2013,0.0709),(2014,0.0678),(2015,0.0647),(2016,0.0614),(2017,0.0606),(2018,0.0597),(2019,0.0499),(2020,0.0495),(2021,0.0495),(2022,0.0471),(2023,0.0448),(2024,0.0427),(2025,0.0406),(2026,0.0386),(2027,0.0368),(2028,0.035),(2029,0.0333),(2030,0.0317)) Unit: %

**Science and technology investment intensity** = WITH LOOKUP (time, ([(2013,0)-(2030,1)],(2013,0.0057892),(2014,0.00541636),(2015,0.00598387),(2016,0.00604428),(2017,0.00716114),(2018,0.00818809),(2019,0.00943969),(2020,0.0107225),(2021,0.0104263),(2022,0.0112602),(2023,0.0121607),(2024,0.0131332),(2025,0.0141836),(2026,0.0153179),(2027,0.0165429),(2028,0.0178659),(2029,0.0192947),(2030,0.0208377)) Unit: %

**Share of environmental pollution control investment** = WITH LOOKUP (time, ([(2013,0)-(2030,1)],(2013,0.00343),(2014,0.00333),(2015,0.00484),(2016,0.00279),(2017,0.00393),(2018,0.00311),(2019,0.00328),(2020,0.00312),(2021,0.00336),(2022,0.00348),(2023,0.0036),(2024,0.00373),(2025,0.00386),(2026,0.004),(2027,0.00414),(2028,0.00429),(2029,0.00444),(2030,0.0046)) Unit: %

**Share of raw coal consumption** = WITH LOOKUP (time, ([(2013,0)-(2030,1)],(2013,0.00171753),(2014,0.00099882),(2015,0.001013),(2016,0.00083645),(2017,0.00037403),(2018,0.00036772),(2019,0.00017085),(2020,0.00010462),(2021,7.367e-05),(2022,5.187e-05),(2023,3.652e-05),(2024,2.571e-05),(2025,1.811e-05),(2026,1.275e-05),(2027,8.98e-06),(2028,6.32e-06),(2029,4.45e-06),(2030,3.13e-06)) Unit: %

**Energy intensity** = WITH LOOKUP (time, ([(760000,0)-(7.5e+06,1)],(763672,0.670535),(757988,0.678732),(883300,0.744122),(952000,0.734212),(1.24028e+06,0.710042),(1.52278e+06,0.661839),(1.8158e+06,0.672622),(2.16279e+06,0.674322),(2.36869e+06,0.675599),(2.69882e+06,0.676879),(3.07497e+06,0.678161),(3.50353e+06,0.679446),(3.99182e+06,0.680733),(4.52662e+06,0.682023),(5.13306e+06,0.683315),(5.82075e+06,0.684609),(6.60057e+06,0.685906),(7.48486e+06,0.687206)) Unit: tons / 10^4^ yuan

**Input factor of logistics industry** = Logistics industry output value / GDP (2013) Unit: dmnl

**Environmental pollution loss factor** =1/10000 Unit: dmnl
