# Supplementary material for: Evaluation and prediction of carbon emission from logistics at city scale for low-carbon development strategy
Source: PLoS One. 2024 Feb 29;19(2):e0298206. doi: 10.1371/journal.pone.0298206 (PMC10903878; doi:10.1371/journal.pone.0298206)
Supplement: S5 File — (DOCX) [file pone.0298206.s005.docx]

**Supplementary Materials**

**5.** **Model calibration**

(1) Operational test

The operation test mainly refers to the sensitivity analysis of the internal variable parameters of the model, which is used to study whether the model is stable under the condition of step change. Taking Suzhou City as an example, the data from 2013 to 2020 are selected for operation test. Under the conditions of Current1, Current2 and Current3, the simulation steps are set to 1,0.5 and 0.25 respectively, and the GDP, emissions are tested. The test results are as Fig S2. The four variables of GDP, output value of logistics industry, total energy consumption and carbon emissions of logistics industry are basically consistent in different step size situations, indicating that the model is overall stable and passes the operational test.


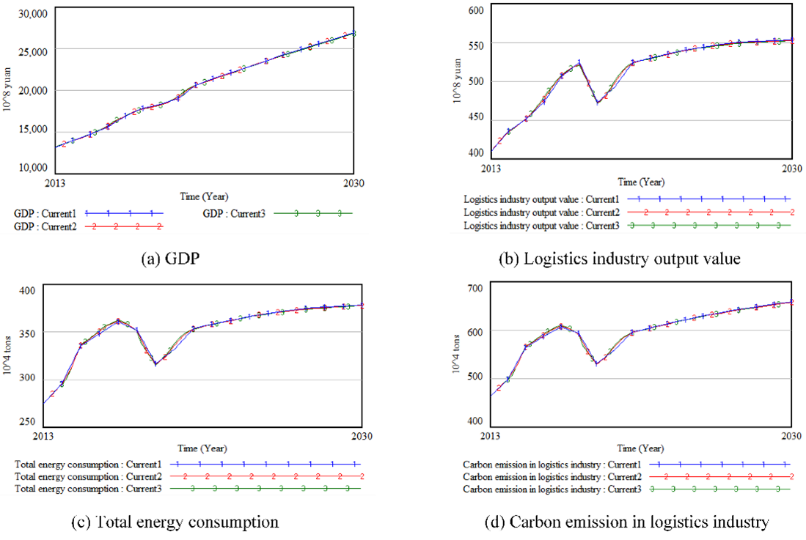


**Fig S2. Simulation results at different step sizes.**

(2) Sensitivity test

Sensitivity test refers to the adjustment of important variables in the model to test whether the relevant variables are sensitive to their changes. For the economic subsystem, we set Current2 to increase the economic growth rate by 5% and Current3 to increase the economic growth rate by 10%, and run the model to obtain the results shown in Fig S3.


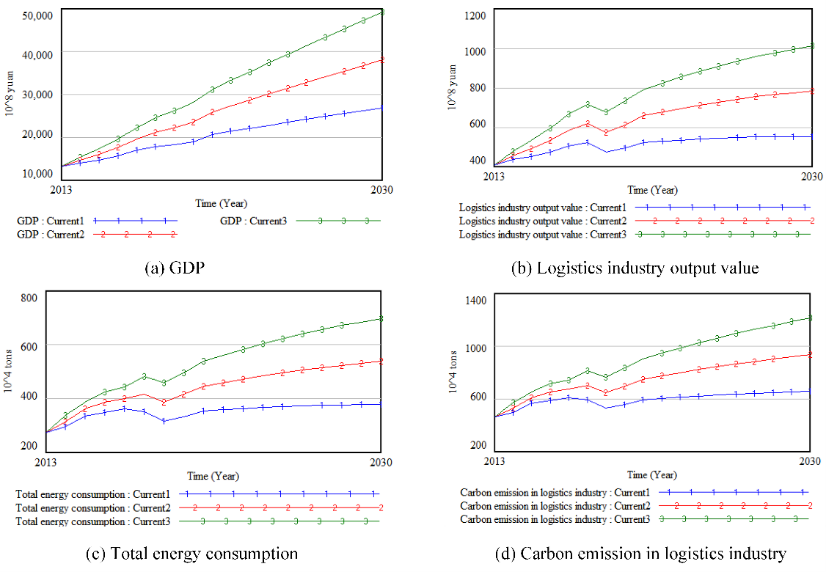


**Fig S3. Sensitivity analysis results.**

With the increase of GDP annual growth rate, GDP, logistics industry output value, total energy consumption and logistics industry carbon emissions increased significantly and the trend remained stable. And as the proportion of GDP annual growth rate increases, the upward trend of the four variables is more obvious. It can be inferred that the system model has good sensitivity and passes the sensitivity test.

(3) Historical test

The historical test is to compare the prediction results with the historical data after the simulation, and observe the similarity between the two. Based on the statistical data from 2013 to 2020, the historical test of carbon emissions in the logistics industry is carried out. The error calculation method is the difference between the predicted value and the historical actual value divided by the historical actual value. The results are shown in Table S3. When the error rate is less than 15 %, the model is effective. It can be seen from Table S3 that the error rate of all prediction results is less than 10 % except for a few cases, which indicates that the simulation effect of the model constructed in this study is good and passes the historical test.

**Table** **S3. Historical examination of the predicted results of LCE in Jiangsu Province (2013-2020).**

| **Year** | **Suzhou** | | | **Nanjing** | | | | | **Wuxi** | | | | |
| --- | --- | --- | --- | --- | --- | --- | --- | --- | --- | --- | --- | --- | --- |
|  | **Actual value /****10^4^ tons** | **Predicted value/10^4^ tons** | **Error rate/%** | **Actual value /10^4^ tons** | **Predicted value/10^4^ tons** | | **Error rate/%** | | **Actual value /10^4^ tons** | | **Predicted value/10^4^ tons** | | **Error rate/%** |
| **2013** | 463.78 | 463.88 | 0.02 | 529.43 | 529.67 | | 0.04 | | 145.68 | | 145.55 | | -0.09 |
| **2014** | 498.84 | 498.88 | 0.01 | 437.40 | 437.60 | | 0.05 | | 158.14 | | 158.03 | | -0.07 |
| **2015** | 567.65 | 564.60 | -0.54 | 491.77 | 484.93 | | -1.39 | | 161.36 | | 159.18 | | -1.35 |
| **2016** | 590.55 | 586.24 | -0.73 | 482.27 | 477.54 | | -0.98 | | 167.05 | | 166.25 | | -0.48 |
| **2017** | 619.34 | 607.13 | -1.97 | 527.71 | 509.00 | | -3.54 | | 175.78 | | 172.53 | | -1.85 |
| **2018** | 607.89 | 593.26 | -2.41 | 498.96 | 480.34 | | -3.73 | | 171.60 | | 169.28 | | -1.36 |
| **2019** | 559.57 | 530.72 | -5.16 | 786.49 | 712.61 | | -9.39 | | 197.27 | | 185.63 | | -5.90 |
| **2020** | 594.40 | 558.54 | -6.03 | 751.09 | 665.83 | | -11.35 | | 203.33 | | 195.15 | | -4.03 |
| **Year** | **Changzhou** | | | **Zhenjiang** | | | | | **Yangzhou** | | | | |
|  | **Actual value /10^4^ tons** | **Predicted value/10^4^ tons** | **Error rate/%** | **Actual value /10^4^ tons** | **Predicted value/10^4^ tons** | | **Error rate/%** | | **Actual value /10^4^ tons** | | **Predicted value/10^4^ tons** | | **Error rate/%** |
| **2013** | 153.26 | 153.31 | 0.03 | 164.44 | 164.47 | | 0.02 | | 80.39 | | 80.38 | | -0.02 |
| **2014** | 162.99 | 162.95 | -0.02 | 167.64 | 167.70 | | 0.03 | | 91.81 | | 91.82 | | 0.01 |
| **2015** | 185.75 | 181.38 | -2.35 | 192.35 | 187.97 | | -2.28 | | 102.84 | | 100.52 | | -2.26 |
| **2016** | 183.97 | 181.83 | -1.16 | 193.95 | 188.44 | | -2.84 | | 105.71 | | 100.79 | | -4.65 |
| **2017** | 202.63 | 200.69 | -0.96 | 198.55 | 201.27 | | 1.37 | | 112.89 | | 105.43 | | -6.61 |
| **2018** | 198.40 | 208.30 | 4.99 | 191.74 | 191.50 | | -0.12 | | 121.15 | | 119.45 | | -1.41 |
| **2019** | 188.95 | 186.58 | -1.26 | 209.99 | 206.36 | | -1.73 | | 96.52 | | 88.72 | | -8.07 |
| **2020** | 193.25 | 176.90 | -8.46 | 216.75 | 210.16 | | -3.04 | | 97.32 | | 87.78 | | -9.81 |
| **Year** | **Taizhou** | | | **Nantong** | | | | | **Huai'an** | | | | |
|  | **Actual value /10^4^ tons** | **Predicted value/10^4^ tons** | **Error rate/%** | **Actual value / 10^4^ons** | **Predicted value/10^4^ tons** | | **Error rate/%** | | **Actual value /10^4^ tons** | | **Predicted value/10^4^ tons** | | **Error rate/%** |
| **2013** | 136.62 | 136.59 | -0.03 | 125.79 | 125.81 | | 0.02 | | 89.44 | | 89.46 | | 0.02 |
| **2014** | 154.23 | 154.24 | 0.01 | 134.60 | 134.64 | | 0.03 | | 103.43 | | 103.41 | | -0.02 |
| **2015** | 172.55 | 170.68 | -1.08 | 153.91 | 152.65 | | -0.82 | | 125.73 | | 123.60 | | -1.70 |
| **2016** | 176.03 | 172.91 | -1.77 | 157.88 | 148.93 | | -5.67 | | 130.11 | | 115.63 | | -11.13 |
| **2017** | 181.63 | 173.66 | -4.39 | 168.10 | 163.68 | | -2.63 | | 134.71 | | 131.77 | | -2.19 |
| **2018** | 176.29 | 169.81 | -3.68 | 164.51 | 154.54 | | -6.06 | | 130.69 | | 123.54 | | -5.47 |
| **2019** | 179.44 | 163.15 | -9.08 | 145.66 | 135.27 | | -7.13 | | 152.87 | | 139.50 | | -8.75 |
| **2020** | 190.64 | 172.41 | -9.56 | 162.05 | 140.56 | | -13.26 | | 158.72 | | 142.31 | | -10.34 |
| **Year** | **Yancheng** | | | **Suqian** | | | | | **Xuzhou** | | | | |
|  | **Actual value /10^4^ tons** | **Predicted value/10^4^ tons** | **Error rate/%** | **Actual value /10^4^ tons** | **Predicted value/10^4^ tons** | | **Error rate/%** | | **Actual value /10^4^ tons** | | **Predicted value/10^4^ tons** | | **Error rate/%** |
| **2013** | 80.83 | 80.80 | -0.04 | 29.69 | 29.69 | | 0.00 | | 638.18 | | 638.26 | | 0.01 |
| **2014** | 87.35 | 87.33 | -0.02 | 28.87 | 28.88 | | 0.03 | | 739.86 | | 739.82 | | -0.01 |
| **2015** | 104.49 | 97.68 | -6.52 | 33.46 | 32.67 | | -2.36 | | 829.50 | | 819.76 | | -1.17 |
| **2016** | 106.45 | 97.03 | -8.85 | 34.00 | 32.91 | | -3.21 | | 836.41 | | 823.82 | | -1.51 |
| **2017** | 114.08 | 105.49 | -7.53 | 37.27 | 35.48 | | -4.80 | | 649.27 | | 643.29 | | -0.92 |
| **2018** | 112.94 | 107.61 | -4.72 | 36.75 | 34.79 | | -5.34 | | 612.00 | | 601.83 | | -1.66 |
| **2019** | 121.81 | 117.82 | -3.28 | 24.24 | 21.78 | | -10.17 | | 616.84 | | 587.23 | | -4.80 |
| **2020** | 127.54 | 120.24 | -5.73 | 25.67 | 23.22 | | -9.52 | | 604.94 | | 616.14 | | 1.85 |
| **Year** | **Lianyungang** | | |  |  |  | |  | |  | |  | |
|  | **Actual value /10^4^ tons** | **Predicted value/10^4^ tons** | **Error rate/%** |  |  | |  | |  | |  | |  |
| **2013** | 105.06 | 105.04 | -0.03 |  |  | |  | |  | |  | |  |
| **2014** | 100.15 | 100.15 | 0.00 |  |  | |  | |  | |  | |  |
| **2015** | 116.18 | 114.41 | -1.53 |  |  | |  | |  | |  | |  |
| **2016** | 118.50 | 108.12 | -8.76 |  |  | |  | |  | |  | |  |
| **2017** | 123.45 | 112.65 | -8.75 |  |  | |  | |  | |  | |  |
| **2018** | 120.52 | 117.37 | -2.62 |  |  | |  | |  | |  | |  |
| **2019** | 165.10 | 157.27 | -4.75 |  |  | |  | |  | |  | |  |
| **2020** | 168.93 | 151.90 | -10.08 |  |  | |  | |  | |  | |  |
