# Supplementary material for: Evaluation and prediction of carbon emission from logistics at city scale for low-carbon development strategy
Source: PLoS One. 2024 Feb 29;19(2):e0298206. doi: 10.1371/journal.pone.0298206 (PMC10903878; doi:10.1371/journal.pone.0298206)
Supplement: S6 File — (DOCX) [file pone.0298206.s006.docx]

**Supplementary Materials**

**6. Comparison of trends between the two scenarios in 13 cities in Jiangsu.**


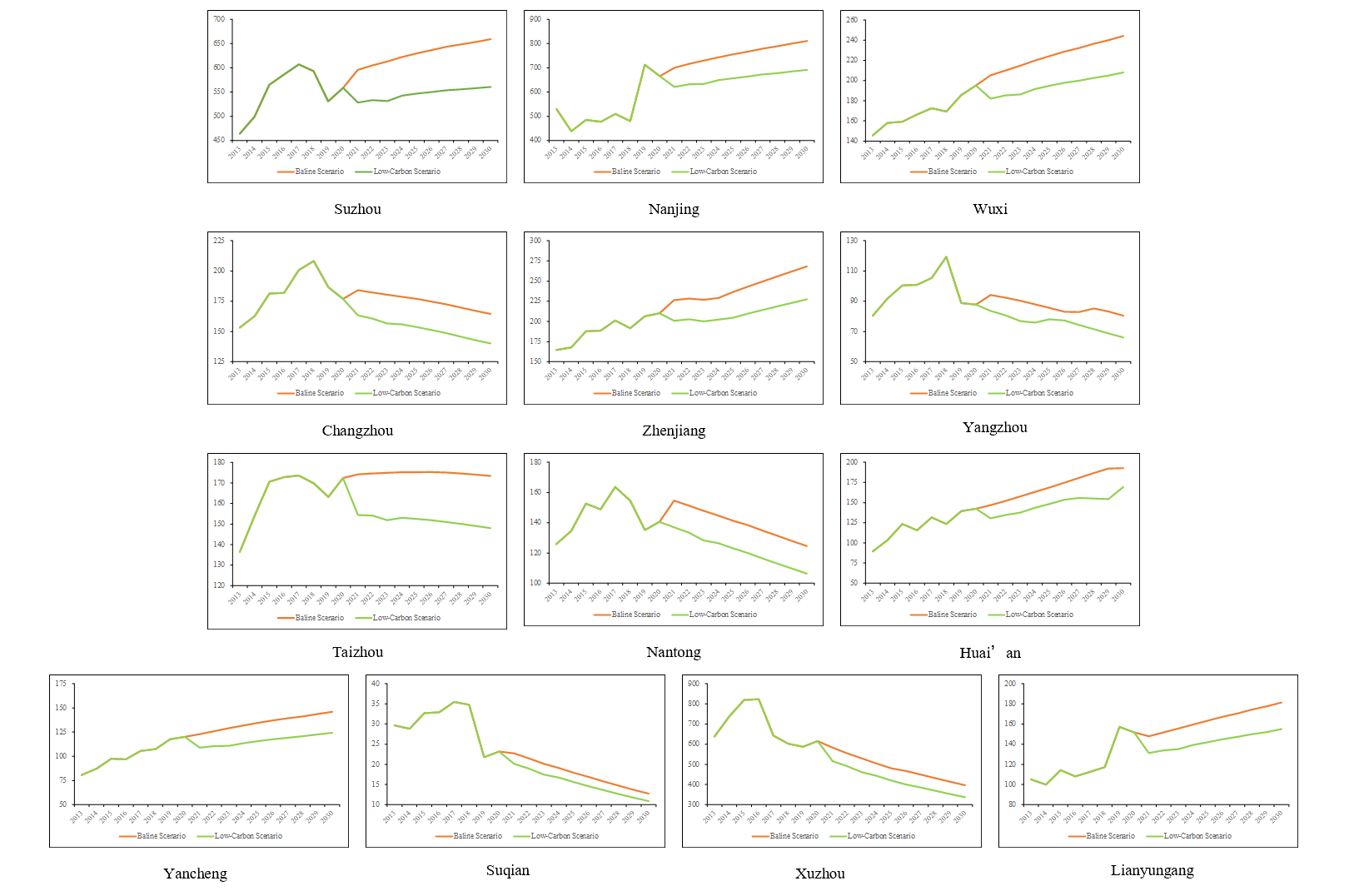


**Fig S4a. LCE under low-carbon scenario (2021-2030)**


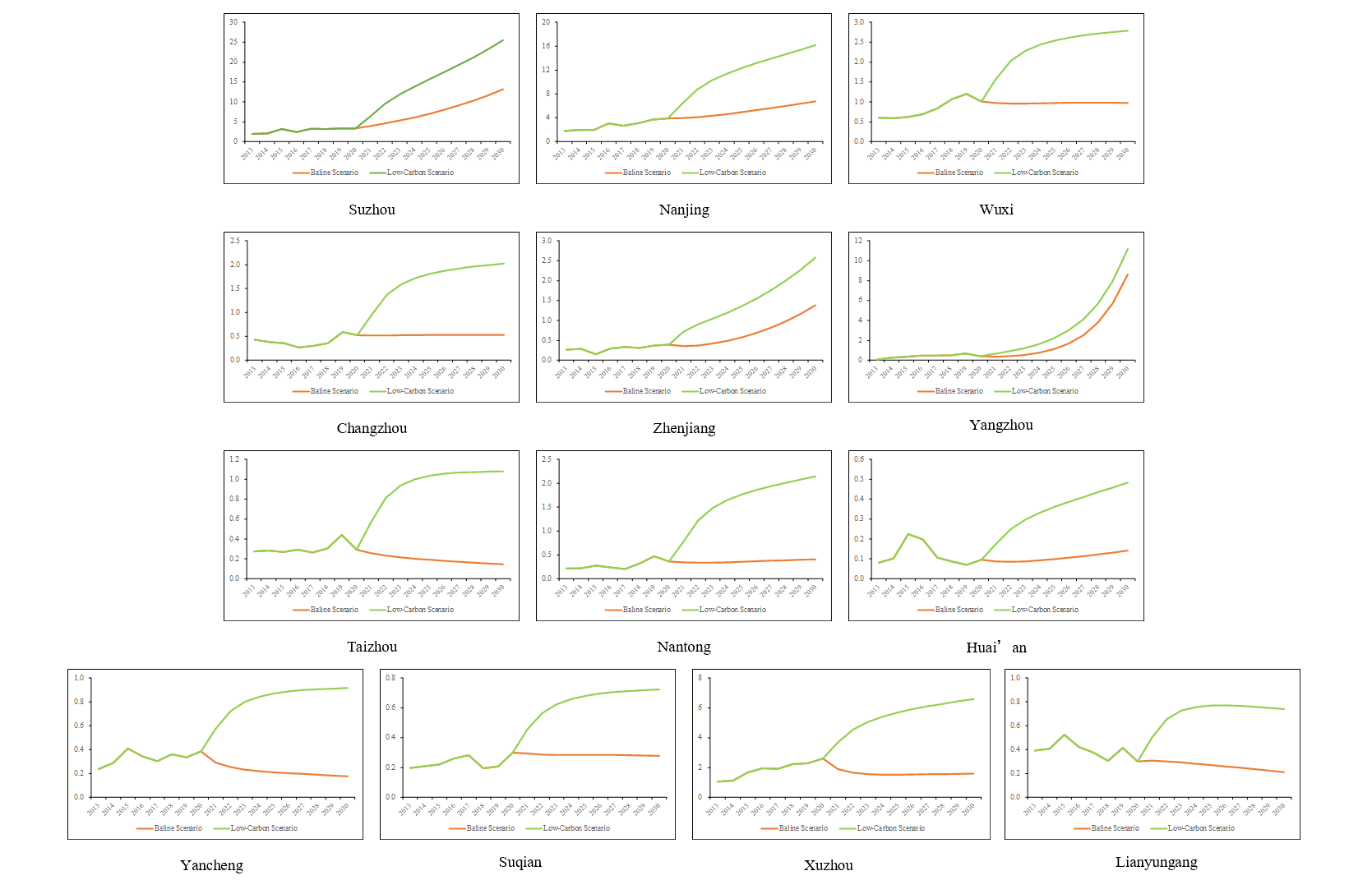


**Fig S4b. The carbon emission reduction of logistics under two scenarios (2021-2030)**
